# Separation and Purification of Fructo-Oligosaccharide by High-Speed Counter-Current Chromatography Coupled with Precolumn Derivatization

**DOI:** 10.3390/molecules23020381

**Published:** 2018-02-10

**Authors:** Wenjuan Duan, Wenhua Ji, Yuanan Wei, Ruixuan Zhao, Zijian Chen, Yanling Geng, Feng Jing, Xiao Wang

**Affiliations:** 1 Qilu University of Technology (Shandong Academy of Sciences), Shandong Analysis and Test Center, Shandong Key Laboratory of TCM Quality Control Technology, 19 Keyuan Street, Jinan 250014, Shandong, China; duanwj4048@126.com (W.D.); jwh519@163.com (W.J.); zhaoruixuan2016@126.com (R.Z.); gengyanling@126.com (Y.G.); jingfeng20052009@163.com (F.J.); 2 Quantum Hi-Tech (China) Biological Co., Ltd., 133 Gaoxin Road West, Hi-tech Zone, Jiangmen 529081, Guangdong, China; yawei@qht.cc (Y.W.); chenzj@qht.cc (Z.C.)

**Keywords:** fructo-oligosaccharide, high-speed counter-current chromatography, precolumn derivatization, separation and purification

## Abstract

High-speed counter-current chromatography (HSCCC) coupled with precolumn derivatization was developed for isolating and purifying fructo-oligosaccharides (FOSs). Firstly, the total FOSs were precolumn derivatized and then separated by high-speed counter-current chromatography (HSCCC) with two-phase solvent system petroleum ether–*n*-butanol–methanol–water (3:2:1:4, *v*/*v*). Secondly, the obtained compounds were deacetylated and the fructo-oligosaccharides (FOSs) with high purity were obtained. Their structures were identified by mass spectrometry (MS) and nuclear magnetic resonance (NMR). This research successfully established a novel strategy for separation and purification of FOS. There is no doubt that the application of the research will be beneficial for the quantitative and qualitative analysis of products containing FOSs.

## 1. Introduction

Prebiotics are ‘non-digestible compounds which through metabolisation by gut microbiota, modulate the compositions and/or activities of the gut microorganisms, thereby conferring beneficial physiological effects on host’ [1]. In recent decades, the bioactivities of prebiotics have attracted more and more attention, worldwide, because of physiological functions such as improving the gut ecosystem, restoring endothelial dysfunction, increasing heme bioavailability, barrier protective function in human intestinal organoids, modulating lipogenesis, reducing triglyceridemia, immunomodulatory and cerebral protective effects [2,3,4,5,6,7,8]. In addition, they could meet the requirements for both sweet tooths and diabetics, due to their non-digestion and non-absorption in body [9].

Fructo-oligosaccharides (FOSs), one of the most significantly used commercial prebiotics in practice, is a mixture (homopolymer) composed of 2–6 fructose monomers connected via β (2–1) glycosidic bonds linked to a terminal glucose residue. In recent years, FOSs have become one of the most important healthy food ingredients, have great potential to improve the quality of many foods, and have been applied in a wide range of fields such as infant milk powder, health care products, pharmaceuticals and cosmetics. Due to the fact that the newer types of functional food ingredients are increasingly popular, high-quality fructo-oligosaccharides (FOSs) are urgently needed to meet the demand for quality control and activity research. To date, a series of methods have been established to purify FOSs, such as fast protein liquid chromatography, size-exclusion chromatography, hydrophilic interaction chromatography, and anion exchange chromatography [10,11,12]. However, due to their low yield, time-consuming processes, and small sample amounts, commercialized reference materials of FOS are expensive. Therefore, it is necessary to develop an efficient method for isolating and purifying FOSs.

High-speed counter-current chromatography (HSCCC) is a support-free liquid-liquid partition chromatographic technique, which has been successfully applied in the isolation and purification of many compounds [13,14,15,16,17]. Compared with many traditional liquid-solid separation methods, HSCCC benefits from a number of advantages: (1) no irreversible adsorption; (2) complete recovery of the injected sample; (3) tailing minimized; (4) low risk of sample denaturation; (5) low solvent consumption; and (6) favorable economics, as there are no expensive columns needed, and only common solvents are required once the initial investment in the instrument has been made [18]. Although there are many significant advantages to HSCCC, it is difficult to apply on FOSs because of their high polar. In addition, to the best of our knowledge, no report on the separation of FOSs by HSCCC has been published. In our previous research, the target compounds were eluted close to each other near the solvent front, even the two-phase solvent system containing n-butanol or salt. In this article, the polarity of FOSs was lowered by precolumn derivatization; then, three FOSs were purified by HSCCC. Their structures were identified by mass spectrometry (MS) and nuclear magnetic resonance (NMR).

The aim of the present work was to establish an efficient method for the separation and purification of FOSs, and to provide a novel strategy for the separation and purification of oligosaccharides.

## 2. Results and Discussion

### 2.1. Strategy for Separation and Purification of FOSs

**Figure 1:**
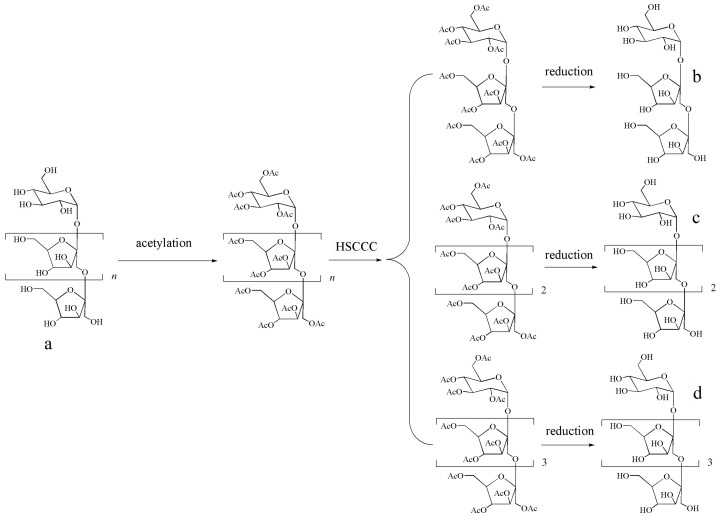
Roadmap of separation of FOSs (**a**) total FOSs; (**b**) the structure of kestose; (**c**) the structure of 1,1-kestotetraose; (**d**) the structure of 1,1,1-kestopentaose.

Because of structural complexity, high polarity, and difficulty of detection, it is difficult to isolate and separate FOSs by traditional methods. In addition, separation is difficult to realize by HSCCC due to their strong hydrophilicity, which leads to the target compounds staying in lower phase, even in n-butanol–water solvent systems. Therefore, a modified strategy for preparative separation of FOSs is needed. Firstly, the polarities of FOSs were lowered by means of acetylation, which was able to satisfy the requirements of separation by HSCCC. Then, the obtained compounds were deacetylated, and the separation of FOSs was realized (the roadmap is shown in Figure 1).

### 2.2. Optimization of HPLC for FOSs

**Figure 2:**
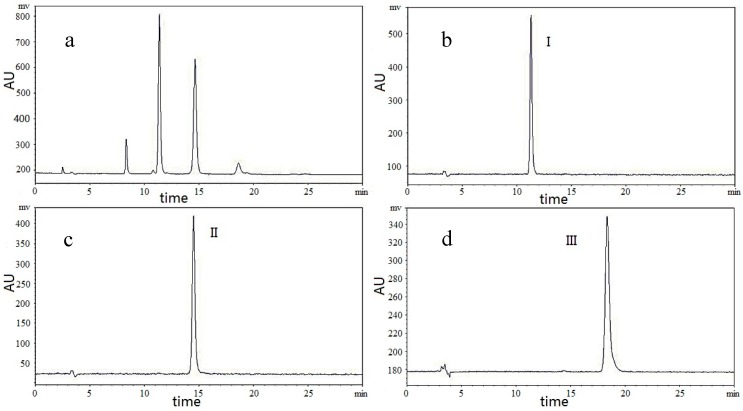
HPLC chromatograms of total acetylated FOSs and separated compounds: (**a**) HPLC chromatograms of total acetylated FOSs; (**b**) HPLC chromatograms of compound I; (**c**) HPLC chromatograms of compound II; (**d**) HPLC chromatograms of HSCCC peak of compound III.

HPLC was used to analyze the total FOSs and purified FOS. According to the structure of the target compounds, three kinds of detectors (DAD, ELSD and RID) were tested. The ELSD was selected due to its high sensitivity and the short time required to achieve system balance. Due to the high polarity of FOSs, the resolution of samples eluted with solvent in C18 chromatographic column is poor. The Xamide 100A and NH_2_ inersil columns were compared, and the Xamide 100A was selected for its good resolution and short processing time. Several elution systems were evaluated, such as isocratic elution of acetonitrile–water and methanol–water. The results showed that the target compounds were able to achieve suitable separation when the mobile phase consisted of acetonitrile–water (70:30, *v*/*v*). The column temperature was 30 °C, the flow rate of the mobile phase was 1.0 mL/min, and the evaporate temperature was 80 °C, the Neb temperature was 90 °C, the gas flow rate was 1.2 SLM. Figure 2 shows that the purities of compounds I, II and III are 98.5%, 98.2%, 98.7%, respectively, as determined by HPLC.

### 2.3. Selection of Precolumn Derivatization Method

In our research, the HSCCC was operated after the precolumn derivatization of FOSs. On suitable method of precolumn derivatization is ether-forming groups, which can be removed easily after completion of the separation process. According to references [19,20,21], the common derivatization methods for hydroxyl groups are methyl, acetyl, and phenyl. Comparing the degree of difficulty of the experiment, the length of operation time, and the degree of hydroxyl group substitutions, acetylation of hydroxyl groups in FOSs was selected.

### 2.4. Selection of Two-Phase Solvent System and Other Conditions of HSCCC

An ideal range of *K*_D_ value for the applied material is a key factor in successfully selecting a suitable two-phase solvent system, which is also the basis for obtaining a good separation effect of the target compounds using HSCCC. As reference [22] describes, a *K*_D_ value in the range of 0.5 to 2 is a requirement for selecting a suitable two-phase solvent system. In this experiment, the structures of FOSs were modified by acetylation. After acetylation, the hydroxyls became acetyls, and their polarities were remarkably reduced. According to the experience of separation of low-polar compounds, a series of solvent systems composed of petroleum ether–ethyl acetate–methanol–water with different volume ratios (1:1:1:1, 4:5:4:5, *v*/*v*), and petroleum ether–*n*-butanol–methanol–water with different volume ratios (3:2:2:3, 3:2:1:4, *v*/*v*) were designed to achieve an ideal *K*_D_ for the target compounds. The evaluated *K*_D_ values for the three compounds are shown in Table 1.

**Figure 3:**
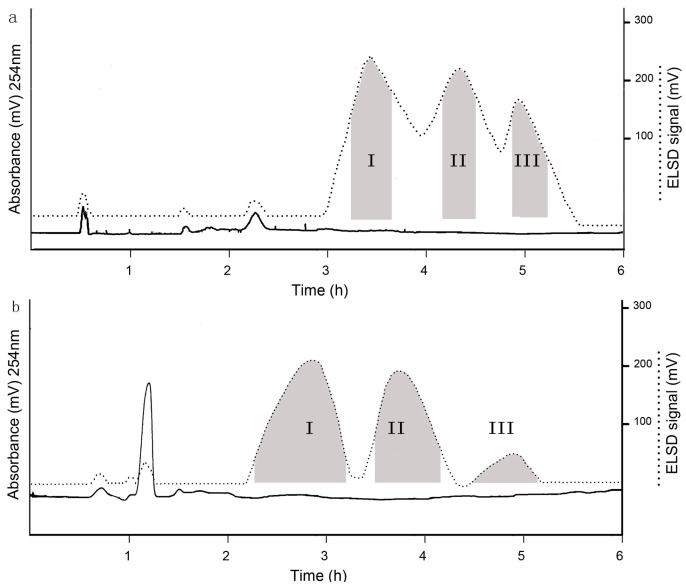
HSCCC chromatogram of the acetylated fructo-oligosaccharides. (**a**) Solvent system: petroleum ether–ethyl acetate–methanol–water (1:1:1:1, *v*/*v*); stationary phase: upper phase; mobile phase: lower phase; flow rate: 2.0 mL/min; revolution speed: 800 rpm; retention of stationary phase: 57.0%; sample load: 200 mg; detection: ELSD; (**b**) Solvent system: petroleum ether–*n*-butanol–methanol–water (3:2:1:4, *v*/*v*); stationary phase: upper phase; mobile phase: lower phase; flow rate: 2.0 mL/min; revolution speed: 800 rpm; retention of stationary phase: 53.0%; sample load: 200 mg; detection: ELSD.

As reference [22] describes, when the *K*_D_ value is smaller than 0.5, the solutes will be eluted close to each other and near the solvent front. As shown in Table 1, the *K*_D_ values of the two-phase solvent systems consisting of ethyl petroleum ether–*n*-butanol–methanol–water (3:2:2:3, *v*/*v*) was smaller than 0.5, which led to bad peak resolution. When the two-phase solvent systems were used for separation, the solutes eluted close to each other and near the solvent front. When the two-phase solvent systems composed of petroleum ether–ethyl acetate–methanol–water (4:5:4:5, *v*/*v*) were used, the *K*_D_ value was greater than 2.0. When the two-phase solvent systems were used for separation, the target compounds were able to be isolated; however, the separation time was too long, which led to excessively broad peaks. The *K*_D_ values of the two-phase solvent systems composed of petroleum ether–ethyl acetate–methanol–water (1:1:1:1, *v*/*v*) and petroleum ether–*n*-butanol–methanol–water (3:2:1:4, *v*/*v*) were between 0.5 and 2, which were suitable for separating the target compounds. Therefore, the HSCCC separation was carried out with the above two solvent systems. When the two-phase solvent system composed of petroleum ether–ethyl acetate–methanol–water (1:1:1:1, *v*/*v*) was used for isolation, compound I, II and III were able to be eluted in a suitable time, but the separation effect of the target compounds was not perfect. As shown in Figure 3, when the two-phase solvent systems composed of petroleum ether–*n*-butanol–methanol–water (3:2:1:4, *v*/*v*) were used, acceptable separation time and good resolution were able to be obtained. The collected fractions of HSCCC were analyzed by HPLC-ELSD. 25.6 mg of compound I, 29.4 mg of compound II, 12.8 mg of compound III with the purities of 98.5, 98.2%, and 98.7%, respectively, were obtained from 200 mg of the crude sample.

## 3. Materials and Methods 

### 3.1. Reagents and Materials

The FOSs were supplied by Quantum Hi-Tech Biological Co., Ltd. (Jiangmen, China).

Ethanol, ethyl acetate, methanol, isopropanol, and petroleum ether (60–90 °C) were all of analytical grade, and were purchased from Jinan Xinhuicheng Chemical Factory, Jinan, China. Acetic anhydride of analytical grade was purchased from China National Pharmaceutical Group Corporation (No. 20 Zhichun Road, Haidian District, Beijing, China). Methanol and acetonitrile of chromatographic grade were purchased from Tedia Company, Inc. (Fairfield, CT, USA), and were used for HPLC analysis. American ultrapure water (18.2 MΩ) was used for all solutions and dilutions, and was obtained with an osmosis Milli-Q water system purchased from Millipore (Bedford, MA, USA).

### 3.2. Precolumn Derivatization

The precolumn derivatization of FOS was conducted by the method described in reference [19]. Acetic anhydride (20.4 g, 0.2 mol) was added to a solution of FOSs (5 g) in pyridine (50 mL) at room temperature. After the reaction mixture was stirred overnight, the solution was concentrated under reduced pressure to obtain the raw product as a yellow syrup, which was used for further purification. 

### 3.3. HSCCC Separation 

#### 3.3.1. Apparatus

Model TBE-300A commercial instrument (Shanghai Tauto Biotech Co., Ltd., Shanghai, China), which comprises a multilayer coil of 150 m in length and 1.6 mm id with a 300 mL total capacity, was employed as the preparative HSCCC instrument in the present study. The β values of this preparative column ranged from 0.5 at the internal to 0.8 at the external (β = r/R, where r is the rotation radius or the distance from the coil to the holder shaft, and R (R = 8 cm) is the revolution radius or the distance between the holder axis and central axis of the centrifuge). A Model HX-1050 constant-flow pump (Beijing Bokang Experimental Equipment Co., Ltd., Beijing, China) was used to pump the solvent into the column. A 30 mL loop manual sample injection (Shanghai Tauto Biotech Co., Ltd., Shanghai, China) was used to inject the sample to the column. Spectra were achieved with a Model 8823A-UV Monitor (Beijing Institute of New Technology Application, Beijing, China) at a wavelength 254 nm. The pH value was monitored by a Model 320 pH meter (Mettler Toledo Instruments, Shanghai, China). The chromatogram was obtained by a portable recorder (Yokogawa Model 3057, Sichuan Instrument Factory, Chongqing, China).

#### 3.3.2. Measurement of Partition Coefficient

The selection of the composition of the two-phase solvent system was based on the distribution constant (*K*_D_) value of the target compounds. HPLC analysis was applied to determine the *K*_D_ values by the following steps [22]: crude extract (5 mg) was dissolved in a test tube (20 mL) to which each phase (5 mL) of the equilibrated two-phase solvent system was added. The capped tube was shaken vigorously for several minutes to thoroughly equilibrate the sample with two phases. Then, 1 mL upper phase and 1 mL lower phase were evaporated separately to dry by the gentle nitrogen stream. The residues were diluted with methanol (1 mL) and analyzed by HPLC to determine the *K*_D_ of the target compound. The *K*_D_ value was calculated by the following equation: *K*_D_ = *A*_U_/*A*_L_ (*A*_U_: the peak area of the upper phase *A*_L_: the lower phase).

#### 3.3.3. Preparation of Sample Solution and Two-Phase Solvent

The crude extract was dissolved in the mixed two-phase solvent system (lower phase and upper phase, 1:1, *v*/*v*) to obtain the sample solution. Each solvent was added to a separatory funnel and then repeatedly and vigorously shaken at room temperature to obtain the two-phase solvent system. Shortly before use, the two phases were separated, following which the upper phase was used as stationary phase and the lower phase was used as mobile phase. 

#### 3.3.4. Separation Procedure

The separation procedure was carried out in accordance with [23]: firstly, the upper phase (stationary phase) was pumped into the multilayer coil column until it was entirely filled at a flow rate of 20 mL/min, while the lower phase (mobile phase) was pumped into the headend of the inlet column at a flow rate of 2 mL/min, and the rotate speed of the apparatus was 800 rpm. After reaching hydrodynamic equilibrium, the sample dissolving a clear mobile phase (200 mg of total FOSs in 8 mL of both phases) was injected into the column via the injection valve. The effluent of the column was continuously monitored with a UV detector at 254 nm, which was collected at 3 min intervals using a fraction collector. After running, the residual solvent in the column was pushed out, and then the retention of the upper phase was measured. The collected fractions were analyzed by HPLC-ELSD. After analysis, the fractions with high purity were collected and dried separately.

### 3.4. Reduction Reaction

The purified acetylated FOSs were reduced by the following method [24]. The target compounds were added to a round-bottom flask while dissolved in MeOH. NaOMe (2 g, 0.037 mol) was added in a single portion and stirred at room temperature for 1 hour. After stirring for 20 min, the reaction was neutralized with Dowex 50WX8 (H^+^ form) and then filtered. The resulting solution was concentrated under reduced pressure till thick oil was obtained. Purification by Sephadex LH-20 column chromatography (eluting with methanol) afforded the target compounds as white powder.

### 3.5. HPLC-ELSD Analysis

The HPLC equipment was an Agilent system, and included an Agilent 1260 TCC, an Agilent 1260 quat pump, an Agilent 385-ELSD (Evaporative Light Scattering Detector), and an Agilent workstation (Agilent, Palo Alto, CA, USA).

The prebiotics and FOSs purified from the HSCCC separation were analyzed by HPLC-ELSD with a Xamide 100A column (250 × 4.6 mm, id) and a column temperature of 25 °C. The mobile phase consisted of acetonitrile–water (75:25, *v*/*v*) at a flow rate of 1.0 mL/min. 

### 3.6. Structural Identification 

The identification of target compounds was carried out by ESI-MS on an Agilent 1100/MSD and by ^1^H-NMR, ^13^C-NMR spectra on a Varian-600 NMR spectrometer with D_2_O solvent and tetramethylsilane (TMS) as internal standard. Comparing the ESI-MS, ^1^H-NMR, and ^13^C-NMR data with the literature, the compounds I–III were identified as 1-kestose, 1,1-kestotetraose, and 1,1,1-kestopentaose, respectively.

*Compound* kestose: ESI-MS *m*/*z*: 527 [M + Na]^+^. ^1^H-NMR (D_2_O, 600 MHz) δ: 5.26 (1H, d, *J* = 3.6 Hz, G-1), 3.38 (1H, dd, *J* = 4.2, 9.6 Hz, G-2), 3.67 (1H, m, G-3), 3.30 (1H, t, *J* = 9.0 Hz, G-4), 3.58 (1H, m, G-5), 3.64 (2H, m, G-6), 3.67 (1H, m, Fa-1), 3.57 (1H, m, Fa-1), 4.11 (1H, d, *J* = 9.0 Hz, Fa-3), 3.87 (1H, t, *J* = 8.4 Hz, Fa-4), 3.71 (1H, m, Fa-5), 3.56 (1H, m, Fb-1), 3.50 (1H, m, Fb-1), 4.02 (1H, d, *J* = 8.4 Hz, Fb-3), 3.91 (1H, t, *J* = 8.4 Hz, Fb-4), 3.70 (1H, m, Fb-5), 3.68 (1H, m, Fb-6), 3.52 (1H, m, Fb-6). ^13^C-NMR (D_2_O, 150 MHz) δ: 92.4 (G-1), 71.0 (G-2), 72.3 (G-3), 69.1 (G-4), 72.4 (G-5), 60.0 (G-6), 60.2 (Fa-1), 103.6 (Fa-2), 76.5 (Fa-3), 73.7 (Fa-4), 81.0 (Fa-5), 62.0 (Fa-6), 60.7 (Fb-1), 103.1 (Fb-2), 76.5 (Fb-3), 74.3 (Fb-4), 81.1 (Fb-5), 62.2 (Fb-6). Compared with the data given in reference [25], peak I was identified as kestose.

*Compound* 1,1-kestotetraose: ESI-MS *m*/*z*: 689 [M + Na]^+^. ^1^H-NMR (D_2_O, 600 MHz) δ: 5.25 (1H, d, *J* = 3.6 Hz, G-1), 3.35 (1H, dd, *J* = 4.2, 9.6 Hz, G-2), 3.57 (1H, m, G-3), 3.29 (1H, t, *J* = 9.6 Hz, G-4), 3.64 (1H, m, G-5), 3.63 (2H, m, G-6), 3.66 (1H, m, Fa-1), 3.57 (1H, m, Fa-1), 4.10 (1H, d, *J* = 9.0 Hz, Fa-3), 3.87 (1H, t, *J* = 8.4 Hz, Fa-4), 3.69 (1H, m, Fa-5), 3.63 (1H, m, Fa-6), 3.61 (1H, m, Fa-6), 3.65 (1H, m, Fb-1), 3.55 (1H, m, Fb-1), 4.05 (1H, d, *J* = 8.4 Hz, Fb-3), 3.90 (1H, t, *J* = 8.4 Hz, Fb-4), 3.68 (1H, m, Fb-5), 3.64 (1H, m, Fb-6), 3.57 (1H, m, Fb-6), 3.57 (1H, m, Fc-1), 3.50 (1H, m, Fc-2), 4.01 (1H, d, *J* = 8.4 Hz, Fc-3), 3.93 (1H, t, *J* = 8.4 Hz, Fc-4), 3.68 (1H, m, Fc-5), 3.65 (1H, m, Fc-6), 3.57 (1H, m, Fc-6). ^13^C-NMR (D_2_O, 150 MHz) δ: 92.4 (G-1), 71.0 (G-2), 72.4 (G-3), 69.1 (G-4), 72.3 (G-5), 60.0 (G-6), 60.9 (Fa-1), 103.1 (Fa-2), 76.6 (Fa-3), 73.7 (Fa-4), 81.0 (Fa-5), 62.1 (Fa-6), 60.7 (Fb-1), 102.9 (Fb-2), 76.6 (Fb-3), 74.2 (Fb-4), 81.0 (Fb-5), 62.1 (Fb-6), 60.2 (Fc-1), 103.6 (Fc-2), 77.3 (Fc-3), 74.3 (Fc-4), 81.1 (Fc-5), 62.1 (Fc-6). Compared with the data given in reference [26], peak II was identified as 1,1-kestotetraose.

*Compound* 1,1,1-kestopentaose: ESI-MS *m*/*z*: 851 [M + Na]^+^. ^1^H NMR (D_2_O, 600 MHz) δ: 5.26 (1H, d, *J* = 4.2 Hz, G-1), 3.37 (1H, dd, *J* = 4.2, 9.6 Hz, G-2), 3.58 (1H, m, G-3), 3.30 (1H, t, *J* = 9.0 Hz, G-4), 3.67 (1H, m, G-5), 3.62 (2H, m, G-6), 3.56 (1H, m, Fa-1), 3.65 (1H, m, Fa-1), 4.10 (1H, d, *J* = 8.4 Hz, Fa-3), 3.86 (1H, t, *J* = 9.0 Hz, Fa-4), 3.57 (1H, m, Fa-5), 3.54 (2H, m, Fa-6), 3.65 (1H, m, Fb-1), 3.71 (1H, m, Fb-1), 4.07 (1H, d, *J* = 8.4 Hz, Fb-3), 3.91 (1H, t, *J* = 8.4 Hz, Fb-4), 3.58 (1H, m, Fb-5), 3.55 (2H, m, Fb-6), 3.55 (1H, m, Fc-1), 3.70 (1H, m, Fc-1), 4.05 (1H, d, *J* = 8.4 Hz, Fc-3), 3.94 (1H, t, *J* = 8.4 Hz, Fc-4), 3.58 (1H, m, Fc-5), 3.55 (2H, m, Fc-6), 3.49 (1H, m, Fd-1), 3.58 (1H, m, Fd-1), 4.01 (1H, d, *J* = 8.4 Hz, Fd-3), 3.94 (1H, t, *J* = 8.4 Hz, Fd-4), 3.58 (1H, m, Fd-5), 3.56 (2H, m, Fd-6). ^13^C NMR (D_2_O, 150 MHz) δ: 92.3 (G-1), 71.0 (G-2), 72.3 (G-3), 69.1 (G-4), 72.4 (G-5), 60.0 (G-6), 60.3 (Fa-1), 103.5 (Fa-2), 76.5 (Fa-3), 73.7 (Fa-4), 81.0 (Fa-5), 62.1 (Fa-6), 60.5 (Fb-1), 103.1 (Fb-2), 76.5 (Fb-3), 74.1 (Fb-4), 81.0 (Fb-5), 62.1 (Fb-6), 60.8 (Fc-1), 102.9 (Fc-2), 77.2 (Fc-3), 74.2 (Fc-4), 81.0 (Fc-5), 62.1 (Fc-6), 60.8 (Fd-1), 102.9 (Fd-2), 77.4 (Fd-3), 74.3 (Fd-4), 81.1 (Fd-5), 62.1 (Fd-6). Compared with the data given in reference [27], peak III was identified as 1,1,1-kestopentaose. 

## 4. Conclusions

The present research indicated that a novel strategy had been developed to separate and purify FOS from total FOSs. Firstly, the sample was structurally modified by acetylation. Then, the acetylated target compounds were isolated by HSCCC with two-phase solvent system composed of petroleum ether–*n*-butanol–methanol–water (3:2:1:4, *v*/*v*), and three compounds with high purity were obtained in one step. Finally, the target compounds were obtained by reduction reaction whose structure was identified by ESI-MS and NMR. In the present research, kestose in 98.5% purity, 1,1-kestotetraose in 98.2% purity and 1,1,1-kestopentaose in 98.7% purity were obtained in a single run. Compared with classic methods such as pre-HPLC, HSCCC coupled with precolumn derivatization is characterized by excellent sample loading, simple operation, and as being less time-consuming and more environmentally friendly. This study indicated that it is an efficient method for the separation and purification of FOSs, and provides a novel strategy for the isolation of oligosaccharides.

## Figures and Tables

**Table 1:** The *K*_D_ values of compound I, compound II, and compound III in different two-phase solvent systems used in HSCCC.

Solvent Systems		*K*_D_ Values		
		I	II	III
petroleum ether–ethyl acetate–methanol–water	1:1:1:1	1.29	1.47	1.61
	4:5:4:5	2.04	2.41	2.93
petroleum ether–*n*-butanol–methanol–water	3:2:2:3	0.28	0.35	0.52
	3:2:1:4	1.34	1.97	2.68
